# Supplementary material for: Quantitative Cross-linking/Mass Spectrometry Using Isotope-labeled Cross-linkers and MaxQuant
Source: Mol Cell Proteomics. 2016 Jun 14;15(8):2769–78. doi: 10.1074/mcp.M115.056481 (PMC4974350; doi:10.1074/mcp.M115.056481)
Supplement: Supplemental Data [file supp_15_8_2769__index.html]

Quantitative cross-linking/mass spectrometry using isotope-labeled cross-linkers and MaxQuant — Quantitative Cross-linking/Mass Spectrometry Using Isotope-labeled Cross-linkers and MaxQuant — QCLMS by Isotope Labeling and MaxQuant — Supplemental Data 

# Quantitative Cross-linking/Mass Spectrometry Using Isotope-labeled Cross-linkers and MaxQuant

## Supplemental Data

- Supplemental Table S1 (.xlsx, 21 KB) - List of quantified cross-linked peptides in data set 2
- Supplemental Table S2 (.txt, 96 KB) - Example input library of data set 2 for MaxQuant (v 1.5.4.1)
